# A Novel Method for Separating Full and Empty Adeno-Associated Viral Capsids Using Ultrafiltration

**DOI:** 10.3390/membranes14090194

**Published:** 2024-09-12

**Authors:** Deepraj Sarmah, Scott M. Husson

**Affiliations:** Department of Chemical and Biomolecular Engineering, Clemson University, 127 Earle Hall, Clemson, SC 29634, USA; dsarmah@clemson.edu

**Keywords:** AAV purification, AAV ultrafiltration, AAV membrane filtration, AAV PCTE membrane, AAV infectivity, AAV live cell imaging, AAV ELISA/qPCR, AAV TEM

## Abstract

Adeno-associated viral vectors (AAVs) are the predominant viral vectors used for gene therapy applications. A significant challenge in obtaining effective doses is removing non-therapeutic empty viral capsids lacking DNA cargo. Current methods for separating full (gene-containing) and empty capsids are challenging to scale, produce low product yields, are slow, and are difficult to operationalize for continuous biomanufacturing. This communication demonstrates the feasibility of separating full and empty capsids by ultrafiltration. Separation performance was quantified by measuring the sieving coefficients for full and empty capsids using ELISA, qPCR, and an infectivity assay based on the live cell imaging of green fluorescent protein expression. We demonstrated that polycarbonate track-etched membranes with a pore size of 30 nm selectively permeated empty capsids to full capsids, with a high recovery yield (89%) for full capsids. The average sieving coefficients of full and empty capsids obtained through ELISA/qPCR were calculated as 0.25 and 0.49, indicating that empty capsids were about twice as permeable as full capsids. Establishing ultrafiltration as a viable unit operation for separating full and empty AAV capsids has implications for developing the scale-free continuous purification of AAVs.

## 1. Introduction

Recombinant adeno-associated viral vectors (AAVs) are fast emerging as the predominant delivery vectors for gene therapies due to their low immunogenicity, non-pathogenicity to humans, and long-term gene expression [1,2,3]. The presence of multiple AAV serotypes responsive to specific cell types enables targeted gene therapies that improve treatment outcomes [2,3]. Recent U.S. Food and Drug Administration Agency (FDA) approvals of AAV-based treatments (Luxturna^®^ for RPE65 mutation-associated retinal dystrophy [4,5,6] in 2017; Zolgensma^®^ for Spinal Muscular Atrophy [7] in 2019; Hemgenix^®^ for Hemophilia B [8] in 2022; and Roctavian^®^ for Hemophilia A [9] and Elevedys^®^ for Duchenne muscular dystrophy [10,11] in 2023) indicate the increasing use and diversity of potential AAV treatments. AAV-based gene therapies are being investigated in at least 200 clinical trials [2,3] for conditions such as neurological, hematological, muscular, and ocular diseases and disorders.

There are critical challenges in producing AAVs to meet commercial and clinical demands while maintaining their affordability [1,12]. The high production costs due to low industrial product yields relative to clinical demand contribute to the high costs of gene therapy and limit the broad applicability of AAV-based gene therapies [1,12,13]. To meet this demand, manufacturers have pursued optimizing the AAV capsid yield for several platforms, including using plasmid expression in human embryonic kidney (HEK293) and Chinese hamster ovary (CHO) cell suspension cultures and baculovirus expression vectors in SF9 insect cells [14,15]. Another production challenge is the presence of capsids that are ineffectively packed with DNA cargo or empty [16,17]. Recombinant AAVs can have a lower packing efficiency than wild-type capsids [18], and a higher lot-to-lot variability of the final product has been observed [16,17]. Empty capsids may account for as much as 90% of all capsids [19], and manufacturers must report the purity of the final product according to FDA guidelines [20]. Besides being an impurity in the final product, empty capsids can competitively inhibit full AAVs [19,21], reducing the product’s efficacy.

Selectively removing the empty capsids produced during capsid assembly without a loss of full capsids is one of the significant challenges to overcome when attempting to improve product yield [12]. Full and empty capsids differ slightly in their mass and charge, which can be exploited by gradient ultracentrifugation, anion exchange, or multimodal metal affinity chromatography. However, the traditional method of using gradient ultracentrifugation to remove empty capsids [22] is challenging to scale and validate when producing large doses of enriched full AAV capsids [23]. Approaches based on anion exchange or multimodal metal affinity chromatography [23,24,25] produce a low AAV capsid yield [25], are slow, and are difficult to operationalize for continuous biomanufacturing. Therefore, there is a pressing need for scale-free separation techniques that achieve high product yields.

Although empty capsids are considered to be impurities and inhibitors of AAV therapies, some studies show they may have therapeutic benefits as decoys to overcome AAV clearance [26,27,28]. Empty capsids bind to neutralizing antibodies produced by the immune system, partially shielding full AAV capsids from the immune response, thereby allowing more functional AAV capsids to reach the target cells. Separation techniques to enrich empty capsids can help to tune the quantity and ratio of full and empty capsids for an effective therapeutic dose.

Separation processes to purify full capsids require a reliable quantification of the full and empty capsids in a final product [25,29,30,31]. A compilation of prevalent quantification methods [29] demonstrates that obtaining the content percentage and titer of the total capsids or genome (implying full capsids) can be based on a single measurement (e.g., optical density and size exclusion chromatography with multi-angle light scattering) or separate measurements of the total capsid titer (e.g., enzyme-linked immunosorbent assay (ELISA), bio-layer interferometry (BLI), flow virometry (FV), static light scattering combined with dynamic light scattering (SLS-DLS)) and genome titer (e.g., quantitative polymerase chain reaction (qPCR), digital droplet PCR (ddPCR), and dye-based binding assays (Dye-BA)). Other methods can provide content ratio information but not the total capsid or genome titer (e.g., transmission electron microscopy with negative stain (TEM), cryo-electron microscopy (cryo-EM), anion-exchange chromatography (AEC), and charge-detection mass spectrometry (CDMS)). However, only the ELISA, qPCR, ddPCR, FV, BLI, and Dye-BA methods are applicable at low concentrations (below 10^12^ capsids/mL). Among these, serotype-specific ELISA, qPCR, and ddPCR are the most used techniques to quantify capsids. BLI is a relatively new technique based on the interference pattern of white light reflected from a layer of immobilized proteins and rivals ELISA with a potentially larger dynamic range. However, measurements require an Octet optical biosensor. FV and Dye-BA may be good options once their robustness and accuracy become well-established.

PCR techniques to quantify the AAV genome may rely on sequences within inverted terminal repeats (ITRs), regulatory elements, or a transgene. Since ITRs are conserved among different types of AAVs, this is considered to be a more universal method. Comparing qPCR and ddPCR, the latter has a smaller coefficient of variation. However, Wang et al. showed that, by using AAVs of known concentrations as standards, the coefficient of variation in qPCR might be comparable to that of ddPCR [32,33].

The reported vector genome may not necessarily report vector potency because of the possibility of non-functional DNA fragments packed in AAVs. Capsid damage may also occur during purification processes due to mechanical or chemical stresses. Therefore, it is essential to measure AAV functionality. Infectivity assays based on transgene expression measured through flow cytometry, plaque detection, or endpoint assays have been used to quantify the relative functionality of AAVs. Live cell imaging is a powerful tool for measuring transgene expression, involving fewer manual steps than flow cytometry and comparable standard deviations for both lentiviruses [34,35] and AAVs [36].

Our approach looks at viral capsid separation using ultrafiltration. Although ultrafiltration has been used extensively for clarifying and concentrating AAVs and other viruses [37,38,39,40], no studies have explored using ultrafiltration for separating empty and full capsids. AAV capsids are speculated to deform under compression to pass through cellular nuclear pores [41], and empty capsids have been shown to deform into ellipsoid shapes through atomic force microscopy [41,42] and selectively pass through nano-pores due to nanopore-induced electro-deformation [43]. Selective permeation through membrane pores based on deformation has been reported to separate supercoiled, linear, and open-circular plasmid isoforms of DNA [44,45,46]. We theorize that the same principle could separate full and empty capsids. Establishing ultrafiltration as a potentially viable unit operation for separating full and empty AAV capsids has implications for developing the scale-free continuous purification of AAV-based gene therapies.

## 2. Materials and Methods

### 2.1. Virus Source and Storage

We procured recombinant AAV2 enriched in full and empty capsids from Virovek, Inc. (Houston, TX, USA) [47]. The full-enriched capsids (product AAV-CMV-GFP) can express green fluorescent protein (GFP) after transduction in mammalian cells. The plasmids used to create empty capsids (product AAV-empty) did not contain any AAV inverted terminal repeats flanking the DNA to be packed into the AAV. The capsid concentrations for full and empty capsid products were reported to be >10^13^ capsids/mL, and we confirmed this through assays (see the following sections). The solution was divided into 10 µL aliquots and stored at −80 °C. Storage in 10 µL aliquots prevented the products from undergoing multiple freeze–thaw cycles. We did not observe any significant changes in the concentrations of the viral capsids after 8 months of cold storage.

### 2.2. Selection of Buffer

Factors related to the stability of AAVs in a buffer, like ionic strength, pH, and the presence of surfactants and free radical oxidation inhibitors, were considered when choosing the working buffer. The aggregation of AAVs is known to be high in pure water, but it decreases and plateaus with an increased ionic strength [48]. The stability of AAVs and preventing adsorption onto surfaces are enhanced by non-ionic detergents like poloxamer 188 (Pluronic F-68) and polysorbate 80 [49]. The presence of ethylenediaminetetraacetic acid (EDTA) mitigates the effects of nucleases, often found at the end of manufacturing processes [49]. AAVs are known to be stable from pH 5.5 to 8.5 and have isoelectric points of 5.9 for full capsids and 6.3 for empty capsids [50].

Our buffer used phosphate-buffered saline (PBS, Fisher Scientific, Waltham, MA, USA) with an ionic strength of 163 mM and 0.1% Pluronic F-68 (Gibco, Waltham, MA, USA) to prevent adsorption onto surfaces. Nine millimolar ascorbic acid (Fisher Scientific) was used to scavenge free radicals and decrease the pH to about 6.0 ± 0.1. One millimolar EDTA (Fisher Scientific) was used to protect the DNA from the effect of nucleases. Our experiments always used a freshly prepared buffer to avoid ascorbic acid degradation.

### 2.3. Determination of Assays

**Figure 1:**
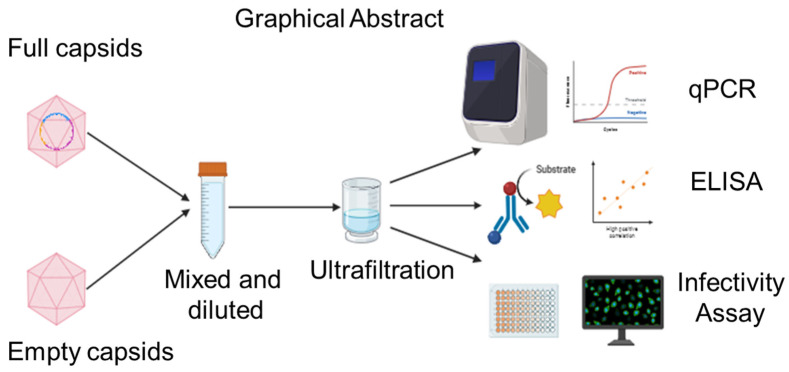
Graphical abstract of experimental protocols. Ultrafiltration of a mixture of full and empty capsids was followed by analysis through qPCR (quantitative polymerase chain reaction), ELISA (enzyme-linked immunosorbent assay), and infectivity assay.

Quantification methods for AAVs are numerous [29,30,31], but many require a relatively high concentration of capsids (>10^12^ capsids/mL). Since dilution in a buffer results in a viral capsid concentration of ~10^10^, our assay options were limited. Figure 1 illustrates the three principal assays used to quantify the total and full/infectious titers of the AAV solutions before and after the separation steps.

#### 2.3.1. ELISA

We used the AAV2-specific ELISA kit (catalog number: PRAAV2R) from Progen Biotechnik (Heidelberg, Germany) to quantify the concentrations of total capsids. The final measurements were made with a Synergy H1 microplate reader (Aligent BioTek, Winooski, VA, USA), and the results were obtained by comparing them with a linear fit of absorbance at 450 nm plotted against concentration.

#### 2.3.2. qPCR

We used qPCR to measure the vector genome number. The primer (sourced from Integrated DNA Technologies, Coralville, IA, USA) was based on ITR sequences (forward: 5-GGA ACC CCT AGT GAT GGA GTT-3; reverse: 5-CGG CCT CAG TGA GCG A-3). AAVs of known concentrations were used as standards (Addgene, Watertown, MA, USA; catalog number 59462-AAV2). Earlier studies established that this approach has fewer steps and less uncertainty than using DNA standards [32,33]. To increase precision, the samples were loaded with an Opentrons^®^ OT-2 automated pipette (Opentrons Labworks, Inc., Long Island City, NY, USA). Real-time measurements were obtained through a qPCR cycler and Bio-Rad CFX Connect (Aligent BioTek) detector.

#### 2.3.3. Infectivity Assay

Parallel to measuring the vector genome, we developed live cell imaging based on the expression of GFP to measure the functional capsids. HEK293T cells (Addgene) were seeded overnight in a 96-well plate, with 150 µL of full-growth DMEM 1Xmedia (Gibco) and 4500 cells per well. Six concentrations were generated through serial half dilutions, starting with 1 mL of an AAV solution with a known vector genome concentration of ~10^10^ capsids/mL (determined by qPCR). Ten microliters of each dilution were added to the wells with the HEK293T cells to generate four replicate wells for each AAV concentration. After 48 h, phase-contrast and fluorescent images were taken of each well using a Cytation 5 imager (Aligent BioTek), keeping the exposure settings constant across the wells. Using the open-source software CellProfiler (v4.2.6 and v4.2.7) [51,52], the fluorescence area and intensity were measured in each well and divided by the corresponding total cytoplasm area. These values were plotted against concentration to generate calibration curves fitted to a power law model.

#### 2.3.4. TEM

Finally, we observed and generated images of the AAV-CMV-GFP (full-enriched) and AAV-empty (empty-enriched) products from Virovek using TEM. A droplet (3 µL) of AAV product was placed on a carbon film-coated TEM grid (Ted Pella, Redding, CA, USA) for 30 s, followed by three rinse steps with 3 µL of distilled water and staining in an aqueous solution of 1% (*w*/*w*) uranyl acetate for 30 s. Imaging was performed at 30,000× in a TEM microscope (HT7830, Hitachi, Tokyo, Japan). The TEM images were analyzed using the CellProfiler software to quantify the full/empty capsid ratio for each product.

### 2.4. Dilution to Desired Ratio and Concentration

Solutions were prepared by mixing 10 µL of full capsid solution and enough empty capsid solution to ensure a 50/50 ratio of full to empty capsids, then diluting the mixture to 25 mL with the buffer. This ensured that the concentration of the capsids after dilution was high enough to be measurable by ELISA. This concentration was also high enough for mammalian cells to express the gene of interest at quantifiable levels for fluorescent imaging.

### 2.5. Ultrafiltration

Ultrafiltration experiments were performed with a 50 mL Amicon^®^ stirred cell (UFSC05001) from MilliporeSigma (Burlington, MA, USA). Commercial membranes were selected with pore sizes close to the AAV capsid diameter measured by TEM. We tested 25 nm (VSWP04700) mixed cellulose ester (MCE) membranes from MilliporeSigma and 30 nm (1270011), 50 nm (PCT0059030), and 80 nm (PCT0089030) hydrophilic polycarbonate track-etched (PCTE) membranes from Sterlitech (Auburn, WA, USA). We selected the MCE membrane for its precise pore size. We included the hydrophilic PCTE membranes due to their low pore size distribution and low protein binding. This range of pore sizes was established in preliminary experiments that found no capsid permeation using the 25 nm membrane and no separation using the 80 nm membrane. Testing commercial membranes made from other polymers was beyond the scope of this feasibility study. We performed ultrafiltration at a pressure of 69 kPa for all the membranes except the 30 nm PCTE, which had a relatively low flow rate, for which we increased the pressure to 172 kPa.

One milliliter of the 25 mL feed AAV solution was collected for concentration measurements. The remaining 24 mL was loaded into the stirred cell, and the magnetic stirrer was set to 300 RPM. The cell was pressurized, and ultrafiltration was performed for a time sufficient to collect 12 mL of permeate. Samples of the permeate and retentate were collected for concentration measurements. We measured the total capsid concentrations of the original feed, permeate, and retentate by ELISA, using the necessary dilutions to bring the concentrations to within the dynamic range of the kit. qPCR was performed for the same diluted samples. A mass balance was used to determine the empty capsid concentration by taking the difference between the total and full capsid concentrations.

## 3. Results

### 3.1. AAV2 Capsids Are Fully Retained by the 25 nm MCE Membrane and Pass Freely through 50 nm and 80 nm PCTE Membranes

Using ELISA, we measured the total capsid concentrations of the full- and empty-enriched capsid solutions from Virovek to be 2.9 × 10^13^ and 6.3 × 10^13^ total capsids/mL. From qPCR, the vector genome concentration of the full-enriched capsid solution was 2.7 × 10^13^, indicating 93% full capsids. The vector genome content of the empty-enriched capsid solution was insignificant (i.e., the concentration of DNA with ITR was <0.01% than that of the full-enriched solution). These data were used to prepare the feed solutions with a 50/50 ratio of full to empty capsids.

**Figure 2:**
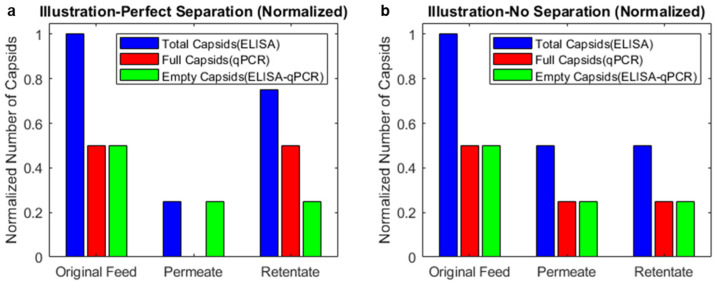
Illustration of hypothetical cases of perfect separation and no separation when half of the feed is passed through the membrane. For perfect separation, all the full capsids are retained in the retentate, and the permeate contains only empty capsids. For no separation, both full and empty capsids are completely permeable, and the permeate and retentate end up having the same composition as the original feed.

Figure 2a illustrates a hypothetical example of perfect separation, in which only empty capsids pass through a membrane. Half the original feed is passed through a membrane in this hypothetical example. The empty capsids pass freely through the membrane, and the full capsids are rejected completely. In this hypothetical example, the permeate contains half the empty capsids, and the retentate contains half the empty capsids and all the full capsids. In the case of no separation (Figure 2b), the full and empty capsids pass freely through the membrane, and there is no difference between the permeate and retentate. Each would contain half the full and empty capsids.

**Figure 3:**
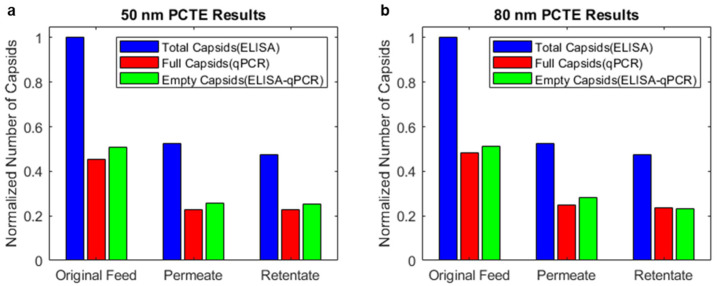
Results from (**a**) 50 nm and (**b**) 80 nm PCTE ultrafiltration experiments. No significant separation of full and empty capsids was observed.

For the 25 nm MCE membrane, the empty and full capsids were fully retained. No capsids were detected in the permeate by ELISA, and the qPCR signal was negligible (<0.1% compared to the original feed). This indicated that we should test membranes with larger pores to enable AAV capsids to pass through the membrane. However, experiments using the PCTE membranes with 50 nm and 80 nm pores showed no separation of full and empty capsids (Figure 3a,b). The recoveries were greater than 90% for the total, full, and empty capsids based on mass balances.

### 3.2. Combining ELISA and qPCR Results Indicates the Enrichment of Empty Capsids in the Permeate and Full Capsids in the Retentate for 30 nm PCTE Membranes

For experiments with the 30 nm PCTE membrane, we observed that the ratio of full capsids in the retentate to the original feed, measured by qPCR, was higher than that of the total capsids, measured by ELISA. This shows an enrichment of full capsids in the retentate. The reverse was true for the permeate (i.e., the ratio of total capsids in the permeate to the original feed was higher than the ratio of full capsids), indicating the enrichment of empty capsids in the permeate.

**Figure 4:**
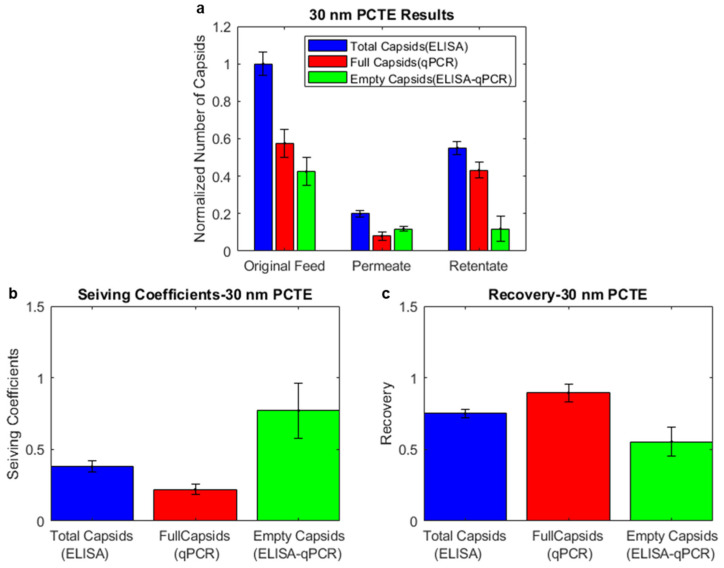
Results from 30 nm PCTE ultrafiltration experiments and analysis through ELISA/qPCR. (**a**) Enrichment of full capsids in the retentate and empty capsids in the permeate were observed, indicating higher permeability of empty capsids. (**b**) Analysis of the sieving coefficients shows that the sieving coefficient of empty capsids is higher than full capsids. (**c**) Analysis of the recoveries shows that the recovery of full capsids is higher than empty capsids.

We combined the results of the ELISA and qPCR to generate Figure 4a. We observed that adding empty-enriched capsids to full-enriched capsids (see Section 2) generally resulted in an original feed that was slightly higher in full capsids, on average, than the expected 50/50 split of full and empty capsids. However, the ratio of full capsids/total capsids in the retentate was always greater than that in the original feed. The permeate had a lower ratio of full capsids/total capsids than the original feed. This shows that 30 nm PCTE membranes are selectively permeable to AAV empty capsids and that AAV capsids may be enriched through ultrafiltration using this membrane.

To analyze these results further, we compared the sieving coefficients of the total, full, and empty capsids. An average sieving coefficient for each experiment was obtained by dividing the permeate concentrations of the total, full, and empty capsids with the averages of the initial concentrations (original feed) and final retentate concentrations. The average sieving coefficients of the total, full, and empty capsids were calculated as 0.38, 0.22, and 0.77, indicating that empty capsids may be about 3.5 times more permeable than full capsids (Figure 4b). The recoveries of the total, full, and empty capsids were calculated as 0.75, 0.89, and 0.55, indicating that more empty capsids than full capsids were lost during the ultrafiltration process.

### 3.3. Combining ELISA and Infectivity Assays also Indicates Enrichment of Empty Capsids in the Permeate and Full Capsids in the Retentate for 30 nm PCTE Membranes

**Figure 5:**
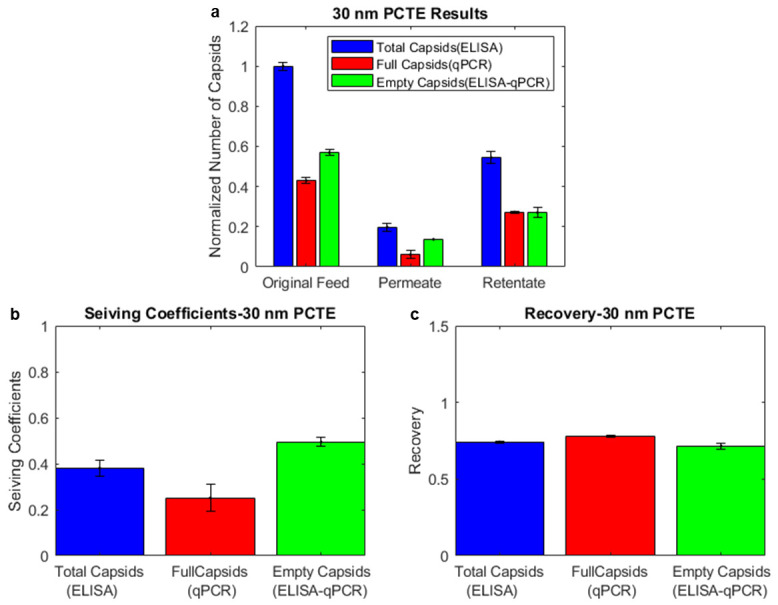
Results from 30 nm PCTE ultrafiltration experiments and analysis through ELISA/infectivity assays. (**a**) Enrichment of full capsids in the retentate and empty capsids in the permeate was observed, indicating higher permeability of empty capsids. (**b**) Analysis of the sieving coefficients shows that the sieving coefficient of empty capsids is higher than full capsids. (**c**) Analysis of the recoveries shows that the recovery of full capsids is marginally higher than that of empty capsids.

We further combined the ELISA and infectivity assay results to validate the enrichment of full capsids in the retentate (Figure 5a). The percentage of full capsids in the original feed was slightly below 50% for the infectivity experiments. However, after ultrafiltration, the proportion of full capsids in the retentate compared to the original feed was always greater than the proportion of total capsids. This validated that the 30 nm PCTE membranes were preferably permeable to empty capsids.

The average sieving coefficients of the total, full, and empty capsids obtained through the ELISA/infectivity assays were calculated as 0.38, 0.25, and 0.49, indicating that empty capsids may be about twice as permeable as full capsids. The enrichment of full capsids in the retentate was lower than that measured by ELISA/qPCR. The possible reasons for this are discussed in Section 4. The recoveries of the total, full, and empty capsids were calculated as 0.74, 0.78, and 0.72, indicating that marginally more empty capsids were lost during the ultrafiltration process.

## 4. Discussion

Several sources manufacture AAVs for laboratory use, including Virovek, Charles River, and Sirion Biotek. Due to their ease of procurement and cost, we used Virovek products derived from an insect-cell-based production process. These insect-cell-based products were initially thought to be indistinguishable from those made using mammalian cells [53]. Ebbernick et al. highlighted some properties of the insect-cell-based AAVs from Virovek compared to other manufacturers. They found differences in the distribution of full versus empty capsids and saw that AAVs produced with insect cells contained a higher proportion of overloaded capsids [54]. Furthermore, the full AAV-GFP-CMV capsids have a vector genome size of ~2.3 kDa, which is lower than the maximum of ~4.7 kDa possible for AAVs. It is possible that a full capsid could pack more than one 2.3 kDa vector genome, which could have implications for the interpretation of assay results.

Supposing that overloaded AAV capsids contain more than one pair of ITR sequences, in this case, we can theorize that, during the separation of full and empty capsids, overloaded capsids and full capsids will be enriched in the retentate. This would cause the qPCR results for the retentate to produce a higher value than expected relative to the original capsids. This could explain the differences between the ELISA/qPCR and ELISA/infectivity assay results, where the ratio of full capsids/total capsids was relatively higher in the qPCR results than in the infectivity assay.

Precautions were taken when formulating the buffer to prevent the adsorption of AAV particles onto the surfaces of the ultrafiltration apparatus and membrane. Nevertheless, we observed higher recoveries (>90%) for the total, full, and empty capsids for the 50 nm and 80 nm PCTE membranes than the 30 nm PCTE membrane. It appeared that some AAV particles were retained within the pores of the membranes, and this effect may have been more significant for the 30 nm membranes due to their similar pore size to the AAVs. Since recovery was higher for the full capsids than the empty capsids, empty capsids may have been more prone to be retained within the membrane pores, which was expected, since a higher proportion of empty capsids entered the pores.

The purity of full capsids after separation by ultracentrifugation can range from 70% to 95%, depending on the precision of the salt gradient and the ability to accurately fractionate the gradient, and the yield of full capsids typically ranges from 50% to 80% [40,55,56,57]. Chromatography-based methods can achieve purities ranging from 80% to 95% for full AAV capsids, and the recovery of full capsids typically ranged from 50% to 90% in some recent studies [23,24,57,58,59,60]. Our recovery measured through qPCR was 89% for full capsids, and the purity increased from an average of 59% to 82% full capsids after one pass. Unlike ultracentrifugation and chromatography, our ultrafiltration method is easy to operationalize for continuous bioprocessing. Unlike antibody-based chromatography methods, our ultrafiltration method can potentially be used across serotypes because of the common size of capsids.

The mechanism of selective permeation is thought to be differences in deformation between empty and full capsids, which has been observed in AFM studies [41,42] and nanopore-induced electro-deformation studies [43]. We theorize that a higher deformation of empty capsids during pressure-driven filtration caused them to have a larger sieving coefficient. Two more potentially exploitable differences between full and empty capsids are differences in hydrodynamic diameter and differences in affinity to certain materials. The reported hydrodynamic diameters of full and empty capsids range from 25 to 40 nm, depending on the measurement method [58,60,61]. Factors like the ionic strength and pH of the buffer may influence these values. Knowing the factors influencing the hydrodynamic diameter may provide insights for size-based separations. Affinity differences between the full/empty capsids and the membrane may enhance separation. Here, again, ionic strength and the presence of Mg^+2^ enhance the affinity differences of full and empty capsids in anion exchange chromatography [20,23,24]. Finally, commercial membranes with a higher porosity than the PCTE membrane used in this feasibility study will increase productivity. This could benefit industrial applications, albeit with the downside that their higher pore size distribution may reduce separation efficiency.

In summary, we report a method for the selective enrichment of full and empty AAV2 capsids based on ultrafiltration. As observed from the infectivity assays, the AAVs retained infectivity after the ultrafiltration process. This makes the method suitable for ultrafiltration–diafiltration strategies for further enrichment and tangential flow filtration (TFF) for processing larger volumes. Transitioning from a stirred cell ultrafiltration setup to a TFF setup is feasible by accounting for differences in flow dynamics, pressure control, and system complexity. Future work is needed to understand the process using one of the many commercial research-scale TFF systems. These TFF systems are highly scalable from the laboratory to pilot to industrial scale.

## Data Availability

The code and data required to generate the figures can be obtained from https://github.com/deeprajs/membranes, accessed on 29 August 2024. Additional data may be provided on request.
